# Enhancement of gallium nitride on silicon (111) using pulse atomic-layer epitaxy (PALE) AlN with composition-graded AlGaN buffer

**DOI:** 10.1038/s41598-023-35677-5

**Published:** 2023-05-31

**Authors:** Marwan Mansor, Rizuan Norhaniza, Ahmad Shuhaimi, Muhammad Iznul Hisyam, Al-Zuhairi Omar, Adam Williams, Mohd Rofei Mat Hussin

**Affiliations:** 1 Low Dimensional Materials Research Centre (LDMRC), Department of Physics, Faculty of Science, Universiti Malaya, 50603 Kuala Lumpur, Malaysia; 2 Silterra Malaysia Sdn Bhd., Lot 8, Phase II Kulim Hi-Tech Park, 09090 Kulim, Kedah Malaysia; 3 MIMOS Berhad, Technology Park Malaysia, 57000 Kuala Lumpur, Malaysia

**Keywords:** Electronics, photonics and device physics, Materials for devices, Nanoscale materials, Materials science

## Abstract

The ability to configure the optimal buffer layer for GaN growth depends on the knowledge of relaxation processes that occurs during the cooling step while countering the tensile stresses due to the contrast of thermal expansion coefficient between GaN and Si(111) substrate. Here, we inaugurate the pulse atomic-layer epitaxy (PALE) AlN layer to reinforce the buffer layer to achieve a thick GaN epilayer which is crucial for high performance power devices. The characteristics of grown GaN on Si substrate based on PALE AlN thickness of 0 ~ 100 nm are investigated along with microstructural evolution between AlN NL and composition-graded AlGaN buffer layer. PALE AlN layer deposited with an optimum thickness of 50 nm and above was observed to exhibit a highly uniform coalesced GaN epilayer surface with root-mean square (RMS) roughness of 0.512 nm. The thickness of the PALE AlN layer substantially affected the crystallinity of the top GaN epilayer where the lowest value for symmetric (0 0 0 2) and asymmetric (1 0 -1 2) x-ray rocking curve analysis were achieved, indicating the reduction of threading dislocation density in the growth structure. Transition of the E_2_ (high) peak from the Raman spectrum shows that the strain compression in GaN epilayer is directly proportional to the thickness of the PALE AlN layer.

## Introduction

In the interest of advantages such as high electron mobility, high breakdown field, wide bandgap (3.4 eV), high electron saturated drift velocity and good thermal stability, gallium nitride (GaN) is considered a crucial choice for applications in high power and high frequency electronic devices, light emitting diode (LED) and laser diodes^1,2^. GaN is generally grown on sapphire, SiC and Si substrates. The same fabrication infrastructure can still be used to achieve this growth on silicon, reducing the requirement for costly production locations and taking advantage of easily available large-diameter Silicon wafers at low cost. In parallel, an improvement in GaN electronic devices on Si substrates seems to conquer many of the major difficulties^3^.

In preceding reports, the growth of GaN on Si structures have focused on strain engineering to compensate for the large extrinsic strain by introducing intermediate layers such as 2-step aluminium nitride (AlN)^4^, aluminium gallium nitride (AlGaN) graded buffer layers (BL)^5^, AlN/GaN strained-layer superlattice (SLS)^6^, and some new methods such as nitridation^7^ and ion-implanting substrate^8^. In order to obtain high-quality crack-free GaN epitaxial films on Si substrates, a high-quality AlN nucleation layer (NL), the very first buffer layer is indispensable. AlN layer can prevent the melt-back etching reaction between Si and Ga atoms, and this capability to initiate the whole epitaxial growth is one of the reasons why AlN is one of the most appropriate materials. Besides, AlN has a smaller in-plane lattice constant (3.112 Å) than GaN (3.189 Å), resulting compressive stress in GaN epitaxial layer^9^. Ultimately, AlN can be grown within the same MOCVD system, which is greatly beneficial to time and cost reduction.

Recently, exceptional works have been reported on the growth of a single-crystalline AlN layer via the pulse atomic-layer epitaxy (PALE) method^10–13^. It was proposed that parasitic reactions between TMAl and NH_3_ precursors can be avoided by the growth of PALE AlN layer while simultaneously assisting the migration of the Al adatoms on the substrate surface. Thus, high quality AlN epilayer with low threading dislocation densities (TDDs) and smooth, flat surfaces can be achieved below conventional growth temperature via conventional MOCVD system^14^. Although most research have been done on the c-plane sapphire substrate, *Altuntas *et al. did investigate the effect of PALE AlN growth temperature on Si(111) on its quality^15^. It concluded that the escalated growth temperature would improve the crystallinity whilst change the growth mode from a 2D-like appearance to a column-like texture. This significant results are the foundation for further enhancements of GaN growth on Si(111) which utilise AlN NL grown at relatively low temperatures.

In this work, we propose a PALE AlN layer sandwiched in between an AlN NL and a composition-graded AlGaN buffer layer, before a thick GaN epilayer on a Si(111) substrate. The influence of various thickness of PALE AlN on the epitaxial surface as well as interface morphology, crystal quality and stress/strain status of GaN epilayer are investigated, by examining these four samples.

## Experimental methods

A 2-inch Si substrate with (111) crystal orientation was employed in this epitaxial growth. The epitaxial process was carried out in SR2000, Taiyo Nippon Sanso MOCVD system with a horizontal reactor with a continuous flow of hydrogen (H_2_) as the gas carrier and nitrogen (N_2_) was utilized as a sub-flow gas to start the growth process on the substrate. Trimethylgallium (TMG) and trimethylaluminium (TMAl) were used as group III precursors, while NH_3_ was used as group V reactant. The cleaning process of the Si substrate was first comprised of an organic cleaning using acetone and isopropanol before ending up with the hydrofluoric dip for 2 min. The substrate was then purged with a nitrogen gun after being rinsed in de-ionized water.

**Figure 1:**
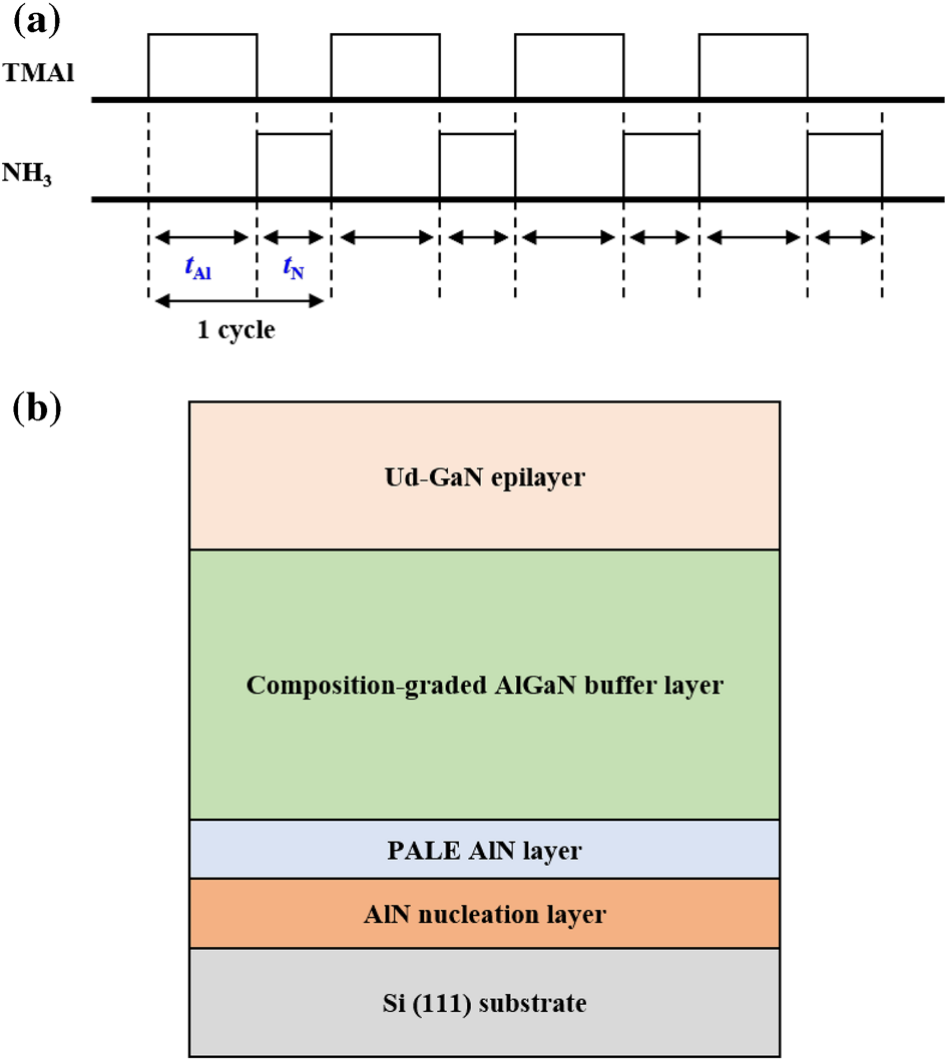
(**a**) The graphical sequence of pulse-flow deposition utilising TMAl and NH_3_, (**b**) the schematic design of the epitaxial growth structure.

There are five steps approach comprises of growing nucleation layer (NL), the compositional-graded AlGaN buffer layer and undoped, ud-GaN. First, the substrate was baked under an H_2_ stream at a temperature of more than 1000 °C in order to eliminate any naturally occurring oxides without causing any deterioration on the substrate surface^16^. Second, an AlN NL approximately 160 nm was deposited whereby the TMAl and NH_3_ sources were flowed simultaneously into the reactor at 1030 °C with growth rate of approximately 0.302 nm/s. Subsequently, an additional AlN buffer layer was grown on top of the AlN NL using the pulse atomic-layer epitaxy (PALE) method at the temperature of 1180 °C. TMAl and NH_3_ were alternately fed into the reactor for *t*_Al_, 4 s and *t*_N_, 2 s, respectively, until the desired thickness was achieved as illustrated in the Fig. 1a. This 4 s window during TMAl supply effectively creates an Al-rich environment, propagating the growth in the c-plane direction as AlN NL layer^17^. The flow of both gases used for PALE was fixed so that the growth rate of the AlN thin film is estimated to be around 1 nm per 1.4 cycles (8.4 s). In order to study the influence of the PALE AlN film thickness on the top GaN epilayer, the PALE cycles were varied to growth PALE layer with thickness of 0 nm (sample A), 25 nm (sample B), 50 nm (sample C) and 100 nm (sample D). Next, a compositional-graded AlGaN buffer of 5 layers with reducing Al composition is grown on top of the AlN layer total up to approximately ~ 620 nm to further counter tensile strain therefore preventing cracks formation on the GaN growth structure^5,18,19^. Finally, during the 5th step, a 500 nm ud-GaN layer was grown at the temperature of 1130 °C and the whole structure of epitaxial film were elucidated in the Fig. 1b. Four samples prepared with PALE AlN’s thickness of 0 nm, 25 nm, 50 nm and 100 nm are labelled as A, B, C and D, respectively.

The surface morphologies of the samples were characterized by Hitachi SU8220 field emission scanning electron microscopy (FESEM) and Park System (NX10 model) atomic force microscopy (AFM). The crystalline quality of the samples was investigated using Rigaku Smartlab high resolution x-ray diffraction (HR-XRD) using *Cu Kα*_*1*_ x-ray target source including x-ray rocking curve (XRC) of symmetric and asymmetric diffractions. Raman (Renishaw inVia Raman microscope) scattering measurement was used to study stress in the films.

## Results and discussion

**Figure 2:**
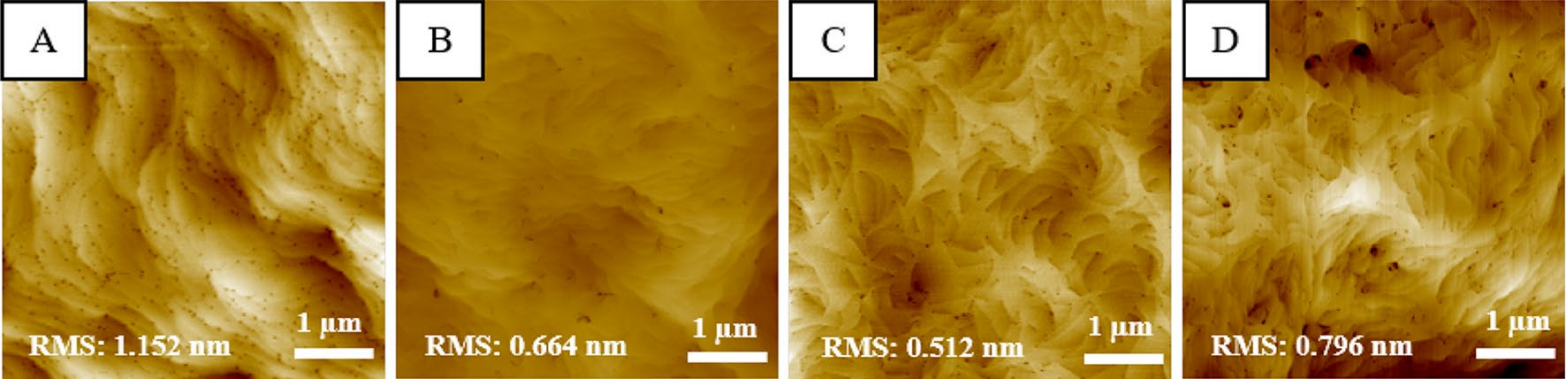
The AFM topography images (image A, B, C and D) 5 µm × 5 µm with the RMS roughness value of the GaN on Si surface morphologies of each sample with PALE AlN buffer layer deposited at various thicknesses.

The surface morphology of GaN on Si structure was successfully ameliorated as the PALE AlN thickness being optimized and the AFM results are shown in Fig. 2a–d. Surface morphology studies for various PALE AlN thicknesses of 0, 25, 50 and 100 nm, are shown with root-mean square surface roughness of samples label A, B, C and D, respectively. It is observed that in sample A, the presence of boundaries with obvious colour contrast showing different depth of surface area. As PALE AlN thicknesses are introduced and increased from sample B to D, the present of boundaries are diminished. Their GaN epilayer surface are fully coalesced and demonstrate a smooth surface. However, sample D with the thickest PALE AlN buffer layer had higher RMS roughness value of 0.796 nm compared to sample C. This is due to formation of rougher surface and cracks as PALE AlN buffer layer already exceeded its critical thickness. Interfaces between AlN and AlGaN graded have experienced partial relaxation, reducing the compressive stress attributed by the AlN nucleation layer. As the whole structure was unable to counter-balance the thermal expansion mismatch, cracks were generated during cooling. The sample with a thicker PALE AlN layer but below the critical thickness provides a better 2-D growth mode, supported by clear step flow and terraces shown by the AFM images. This observation is proven by the RMS surface roughness value measured, where samples A, B and C RMS values are 1.152, 0.664 and 0.512, respectively. This shows an improvement in surface quality by almost 35% with the utilization of PALE AlN. The diminished boundaries by the introduction of PALE AlN can be attributed by the effective 4 s interval in NH_3_ flow encourages more Al adatoms to accumulate resulting in the increase of island grain size and the decrease in boundary density^11^. At a higher thickness of PALE AlN layer, as the grains begin to coalesce, more boundaries diminish and its density decreases steadily. Consequently, this leads to a near atomically-flat surface or smooth surface of the AlN layer. Hence the abrupt interfaces among the AlN NL and composition-graded AlGaN buffer layer can be achieved.

**Figure 3:**
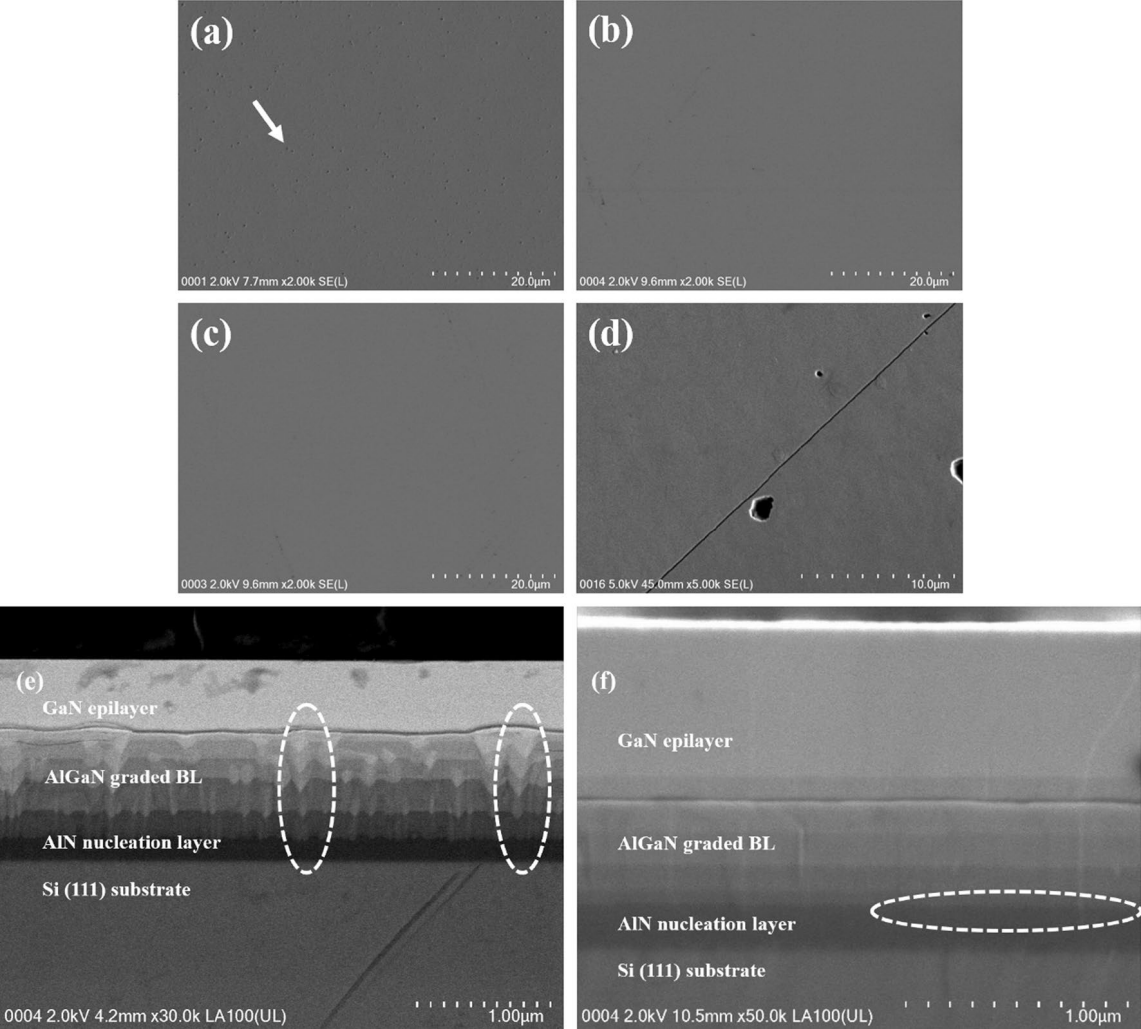
FESEM surface images of sample (**a**) A, (**b**) B, (**c**) C and (**d**) D. FESEM cross-sectional image of sample (**e**) A, and (**f**) C focusing on the interfaces between PALE AlN buffer layer and composition-graded AlGaN buffer layer.

In order to further analyse the interfacial and structural property of GaN/Si hetero-interface grown with various PALE AlN thickness, cross-sectional FESEM measurements are employed. Figure 3a–d shows the image of GaN film surfaces for samples A, B, C and D, respectively. Upon growing the ud-GaN epilayer at temperature of 1130 °C, samples A (without PALE AlN layer) is having pits shown by arrow in Fig. 3a, while the surface of sample B and C (both with PALE AlN layer) features a lateral 2-D growth. It is established that sample A, B and C as in Fig. 3a–c shows a crack-free surface with a suave transition to a smoother surface. Sample B and C also show a crack-free, smooth surface proving the benefit of the PALE method growing AlN layer to produce a crack-free surface while sample D shows cracking at the centre of the wafer as in Fig. 3d. Owing to significant mismatches of thermal expansion coefficient and lattice constant between Si and GaN, it can be seen clearly that surface of the sample D has developed crack networks, possibly due to compressive stress generated by the 100 nm thickness of PALE AlN layer was not being able to compensate the tensile stress entirely.

From the cross-sectional view, Fig. 3e clearly suggests a cause of the pit formation on surface sample A in Fig. 3a. A rough surface of AlN NL grown directly on Si substrate induces a fluctuation in the interfaces among the AlN NL and composition-graded AlGaN buffer layers. We anticipate that these ‘v-shaped’ remained during the growth of the remaining composition-graded AlGaN buffer layer and carries threading dislocations (TDs). Lateral growth was massively affected by this phenomenon which induces the formation of surface defects, namely v-pits. The ‘v-shaped’ pattern seems to be almost similar to the one where particles originate from the AlN NL regrowth interface, which imply that the particles fell onto the substrate before the entire structure grown^20^. Different research showed that large v-shaped defects in strained-layer superlattice (SLS) have been correlated with large gate leakage current in the HEMT structure^21^. Thus, the PALE AlN layer is an alternative method to mitigate the fluctuation in the interfaces between the AlN NL and composition-graded AlGaN buffer layers, as shown in Fig. 3f, which could potentially decrease leakage current in the device’s vertical direction.

Two different planes which are (0 0 0 2)-symmetric scans for screw dislocation study and (1 0 -1 2)-asymmetric scans for mixed-edge rocking curve profiles of the deposited GaN film were obtained with a Rigaku Smartlab high resolution x-ray diffraction (HR-XRD) using *Cu Kα*_*1*_ x-ray target source (λ = 1.5406 Å)^22^. From the results of the XRC analysis, we can estimate the threading dislocation densities (TDDs) for screw (D_S_) as well as mixed-edge (D_E_) using^23^,

1 $${\mathrm{D}}_{\mathrm{S}} =\frac{{\beta {\text{s}}}^{2}}{4.35|{b{\text{s}}|}^{2}},$$

2 $${\mathrm{D}}_{\mathrm{E}}=\frac{{\beta {\text{e}}}^{2}}{4.35|{b{\text{e}}|}^{2}}.$$

Generally, *b*_s_ and *b*_e_ are the Burgers vector sizes of the screw and mixed-edge threading dislocations, respectively, while *β*_s_ and *β*_e_ being the full width at half-maximum (FWHM) values in radians. Here, $b{\text{s}}$ = 0.4982 nm, $b{\text{e}}$ = 0.3112 nm, and the values of the D_S_ and D_E_ are given in Table 1.

**Table 1:** FWHM of XRC measurement and TDDs at various cycle number of PALE AlN buffer layer.

PALE AlN thickness (nm)	5 μm × 5 μm RMS roughness (nm)	5 μm × 5 μm peak-to-valley (nm)	FWHM (002) (arcsec)	FWHM (102) (arcsec)	D_S_ (cm^−1^)	D_E_ (cm^−1^)
A (0 nm)	1.15	11.35	837.36	1425.60	1.53 × 10^9^	1.13 × 10^10^
B (25 nm)	0.66	7.20	813.24	1230.12	1.44 × 10^9^	8.44 × 10^9^
C (50 nm)	0.51	6.58	782.28	1141.20	1.33 × 10^9^	7.27 × 10^9^
D (100 nm)	0.796	9.92	795.60	1186.20	1.38 × 10^9^	7.85 × 10^9^

RMS roughness value from AFM analysis of 5 μm × 5 μm are included as reference.

**Figure 4:**
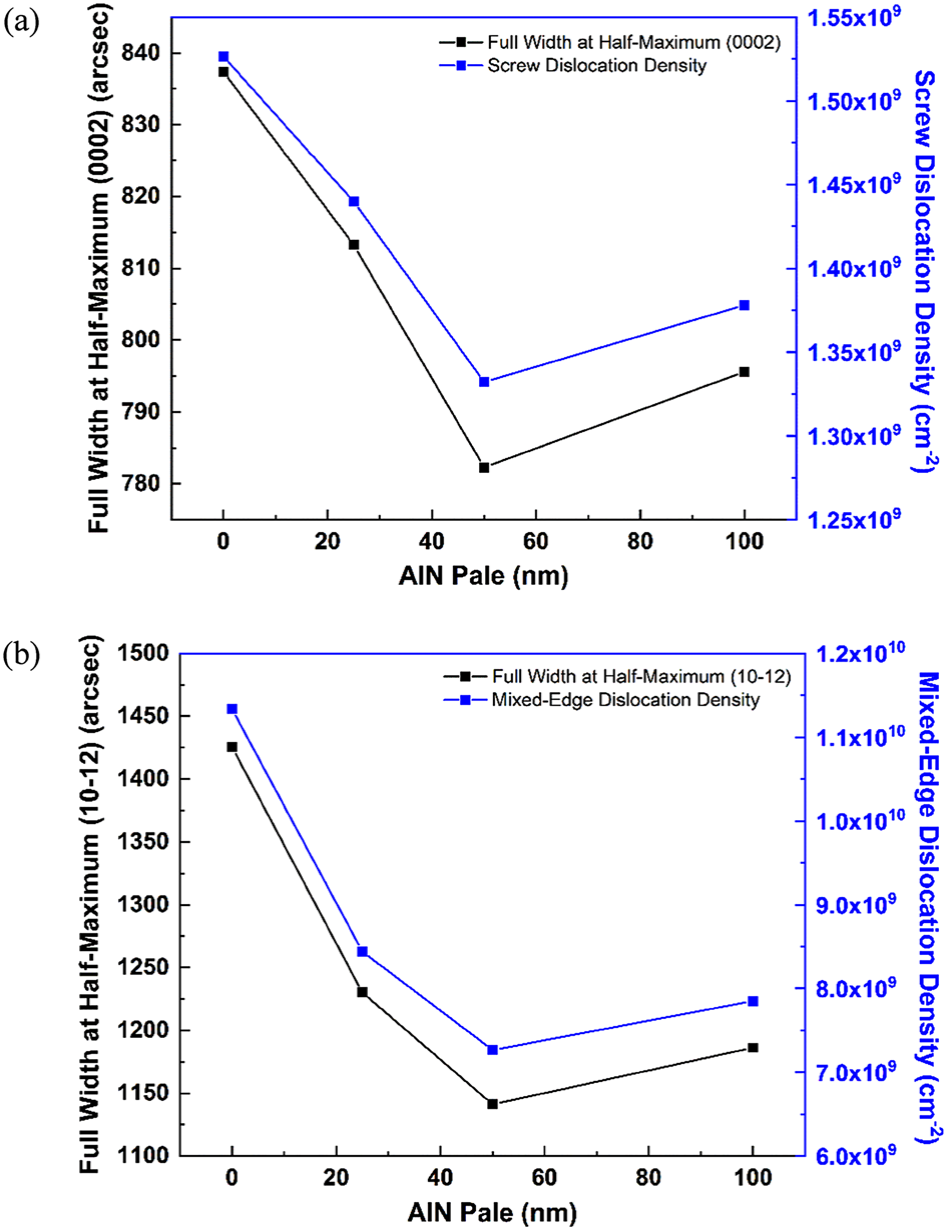
(**a**) FWHM (0 0 0 2) with screw threading dislocation and (**b**) FWHM (1 0–1 2) with mixed-edge threading dislocation, as a function of PALE AlN thickness.

FWHM and dislocation density values of screw and mixed-edge from Table 1 are graphically plotted in Fig. 4a and b, respectively. Both full width at half-maximum (FWHM) trend relative to threading dislocation densities (TDDs) trend attributed by varied PALE AlN thickness. FWHM for sample A without PALE AlN has a broader FWHM compared to all samples with PALE AlN which have relatively narrower FWHM. Here, the crystalline quality of the top GaN films for symmetric (tilt-related) and asymmetric (twist-related) analysis also shows significant improvement with the promotion of PALE AlN thickness, in compatibility with the AFM results. At PALE AlN thickness of 50 nm (sample C), the lowest FWHM for symmetry and asymmetry analysis was discovered, with both values of 782.28 arcsec and 1141.20 arcsec, respectively. Nevertheless, the lowest screw dislocation densities, D_S_ and mixed-edge dislocation densities, D_E_ also calculated 1.33 × 10^9^ cm^−1^ and 7.27 × 10^9^ cm^−1^, respectively. Both dislocations of sample C were cut down by 13% ~ 35% compared to sample A. As it was anticipated, with the low growth rate of PALE technique, the crystalline quality of AlN layer is generally higher than those using higher growth rates^24^. There is only slight difference in the screw and mixed-edge dislocations between sample C and D.

The reduction in threading dislocation density might be in reference to the low RMS roughness as observed in AFM and FESEM characterizations. The smoother interface originated by the PALE AlN layer reduces the v-shaped pattern on first composition-graded AlGaN buffer layer, hence also smoothen up the interface between other buffers and GaN layer. Thus, threading dislocation densities in the epitaxial layers can be reduced. Sample C as reference with lowest FWHM, we regrow the same structure with an alteration (thicker composition-graded AlGaN buffer layer) and managed to achieve 669.96 arcsec and 1108.08 arcsec for symmetry and asymmetry, respectively, for the top GaN epilayer. Sample D experienced a slight increment of threading dislocation which perhaps supported the crack surface phenomenon observed before. The crystal quality of GaN layer XRC analysis pattern showed a good congruence with the AFM and FESEM discussion.

**Figure 5:**
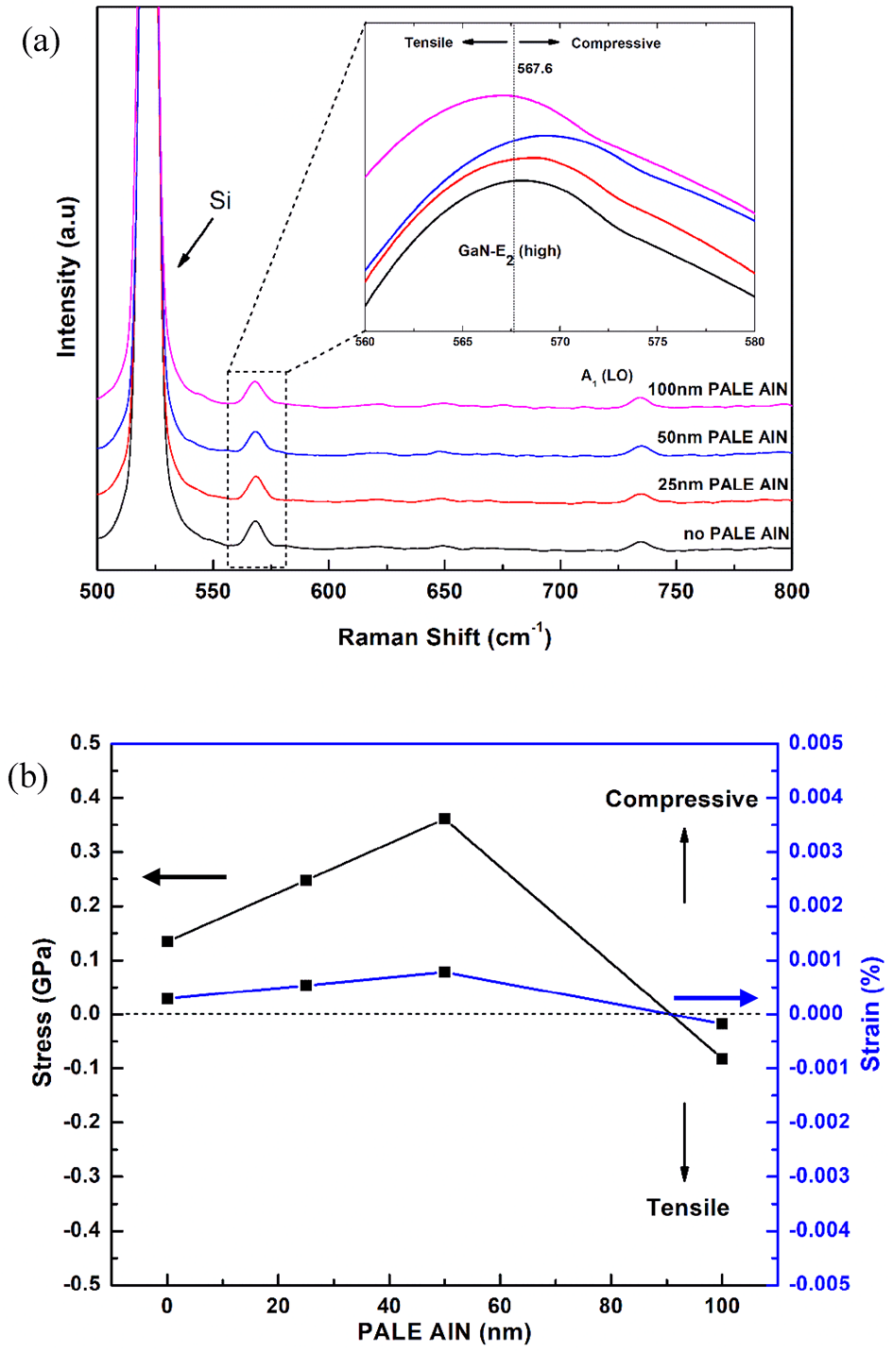
(**a**) Raman spectre of GaN/Si samples to different PALE AlN thickness 0 ~ 100 nm. (**b**) In-plane stress and strain versus PALE AlN thickness.

The strain in the top GaN films was then scrutinized by analysing the shift of the E_2_ phonon mode of the samples with a Renishaw In-Via Raman spectrometer with Ar + laser at a wavelength of 514 nm, as presented in Fig. 5a. As it is known, the E_2_ mode is sensitive to biaxial stress^25^, and the E_2_ mode is located at 567.6 cm^−1^ in bulk GaN as the natural state used for stress measurement^26^. For samples A, B, C and D, the E_2_ phonon mode for GaN is located at 568.2 cm^−1^, 568.7 cm^−1^, 569.2 cm^−1^ and 567.3 cm^−1^, as shown in Fig. 5a respectively.

The in-plane stress, $\sigma$ can be calculated using the equation, $\sigma =\Delta \lambda /\kappa$, where $\Delta \lambda$ is the phonon peak shift and $\kappa$ is the pressure coefficient, 4.3 cm^−1^ GPa^−1^, Ref.^27^ while the in-plane strain, *ε* can be calculated using the equation, $\varepsilon =\sigma /\Upsilon$ where *σ* is the in-plane stress and ϒ is the biaxial stress of GaN, 463 GPa^28^. In-plane stress calculated for each samples A ~ D is 0.13, 0.25, 0.36 and -0.08 GPa respectively as shown in Fig. 5b. There appears to be 0.6 cm^−1^ shift towards higher frequency of E_2_ phonon mode occurs in sample A; similarly, 1.1 cm^−1^ shift and 1.6 cm^−1^ towards higher frequency of E_2_ phonon mode in sample B and C, respectively.

Therefore, there is appropriate amount of compressive stress in GaN epilayer well proportional to the thickness of PALE AlN layer. In contrast, the E_2_ phonon mode for sample D happens to shift slightly 0.3 cm^−1^ towards lower frequency in the peak frequency, exhibit tensile stress in the top GaN layer. This may be due to the deposited AlN film via the PALE technique, was entirely flat and smooth. In addition, the extra thickness has made AlN NL experience partial relaxation, hence, diminishing some compressive stress towards the whole structure. In general, cracks in GaN film occur under biaxial tensile stress owing to the large difference in thermal expansion coefficients of GaN and Si. This occurrence acts as the supporting evidence along with the AFM, FESEM and XRD analysis results, which the PALE AlN layer is able to confine the compressive strain induced from the lattice and thermal mismatch between the Si(111) substrate, AlN epilayer, composition-graded AlGaN buffer layer and latter GaN epilayer, even after the cooling to room temperature.

AlN nucleation layer indeed provides compressive strain due to lattice mismatch and compensate tensile stress from substrate especially during cooling process. AlGaN buffer layers are the main medium to filter threading dislocations. The insertion of PALE AlN buffer layer between AlN nucleation layer and composition-graded AlGaN buffer enhances the interfacial layers. The following describes the relationship between the parameters of the PALE AlN buffer layer and the crystalline quality of GaN epitaxial films. Threading dislocations in the AlN nucleation layer would propagate through the succeeding films, which are composition-graded AlGaN buffer layer, lowering the quality of as-grown films. As a result, enhancement in the crystalline quality of the AlN nucleation layer layer can reduce the initial TDDs, resulting in a reduction in propagating TDs in the subsequent GaN epilayer, thus improving the crystalline quality of GaN epitaxial films. The flat surface of the PALE AlN buffer layer, on the other hand, can encourage the lateral expansion and coalescence of nucleation islands in succeeding films, resulting in a larger volume of defect-free columnar domains, and therefore improving the crystalline quality of following films^29^. In general, a high-quality AlN buffer layer with a flat surface is crucial for improving the crystalline quality of GaN epitaxial films on Si substrates. In contrast, as PALE AlN exceeds critical thickness, it experiences partial relaxation. The elevated tensile stress from PALE AlN buffer layer at 100 nm thickness leading to generated cracks on the topmost GaN epilayer grown on Si(111).

## Conclusion

The extra AlN films were deposited on AlN NL by pulse atomic-layer epitaxy with different numbers of pulse cycles via MOCVD system. The homogeneity of deposited PALE AlN epilayer is critically dependent on the film thickness. Properties of GaN film on Si(111) with different PALE AlN thickness were investigated accordingly. Field emission scanning electron microscopy indicated a highly uniform and abrupt interface between AlN NL and composition-graded AlGaN buffer layer which offers the latter crack-free GaN epilayer grown over the whole structure. The interfaces between AlN NL and composition-graded AlGaN buffer layers are improved with a smooth and abrupt structure which eliminates v-shaped interfacial fluctuation which possibly contribute to leakage current during device’s operation. Root means square roughness decrease to 0.679 nm, while FWHM value of (0002) and (10–12) reduced to 782.28 arcsec and 1141.20 arcsec, respectively with diminished TDDs when PALE AlN thickness were increased to 50 nm. Furthermore, in-stress and in-strain are elevated parallel with Raman spectroscopy shifting towards higher frequency. However, as PALE AlN thickness surpass the critical thickness, the confined compressive stress in GaN epilayer can no longer counter-balance the thermal expansion mismatch-related tensile stresses during temperature cool-down process. In summary, a relatively high-quality crack-free GaN epilayer on Si(111) can be produced by the insertion of PALE AlN layer.

## Data Availability

The data used to support the findings of this study is confidential. Please contact Dr Ahmad Shuhaimi (shuhaimi@um.edu.my) for data request from this study.
